# 0356. Effect of the neuroprotective p53-inhibitor pifithrin-µ in a rodent cardiac arrest model

**DOI:** 10.1186/2197-425X-2-S1-P22

**Published:** 2014-09-26

**Authors:** D Springe, A Putzu, P Zuercher, D Grandgirard, S Leib, SM Jakob, J Takala, M Haenggi

**Affiliations:** University of Bern - Inselspital, Dept. of Intensive Care Medicine, Bern, Switzerland; University of Bern, Institute for Infectious Diseases, Neuroinfection Laboratory, Bern, Switzerland; Federal Office for Civil Protection, Biology Division, Spiez Laboratory, Spiez, Switzerland

## Introduction

Experimental studies have shown that pifithrin-µ has neuroprotective properties by inhibiting the tumor suppressor p53, which is a key regulator of apoptotic cell death [1]. So far no animal experiments with pifithrin-µ have been carried out in the field of cardiac arrest (CA).

## Objectives

We hypothesized that treatment with pifithrin-µ inhibits delayed neurodegeneration in our 8 min CA model.

## Methods

8-min asystolic CA (intravenous KCl in esmolol) was induced in 80 male Wistar rats. 5 rats died during the operation/CA procedure, the remaining 75 were randomized into 2 main groups: control (c) (8-min CA, 10ml/kg solvent (4% dimethylsulfoxamide/phosphate buffered saline) ip after ROSC) and pifithrin (p) (8-min CA, 8mg/kg pifithrin-µ in 10ml/kg solvent ip). 1 animal (p) died after 48 hours. 12 c + 11 p animals were euthanized on day 1, 11 c + 11 p on day 5 and 12 c + 11 p after day 10. 6 Sham operated animals were euthanized on day 1 (n=3) and after day 10 (n=3), but not included into statistics.The rats were assessed daily from preoperative to day 5 and again on day 10 by a behavior score for rats, a neuro-deficit score and a tape-removal-test. On day 0, 4 and 5 the locomotor activity was recorded in an open field test and after day 10 the remaining rats were tested for learning capacities in the water maze experiment. Harvesting of brain for histology of the hippocampus cornus ammonis segment CA1, assessed with cresyl violet (CV) and Fluro-Jade (FJ) staining, was performed on day 1 and day 5.

## Results

After a single dose of 8 mg/kg pifithrin-µ or solvent virtually no apoptosis could be detected after 24 hours in each group. On day 5, we found a trend towards a decreased number of pyknotic cells (CV staining), a tendency towards a preserved hippocampal cell layer and a trend towards less neuronal degeneration (FJ staining) in the (p) compared with the (c) group (see table 1).

**Table 1:** Histology (day 5)

	pifithrin (n=11)	control (n=11)	p
Number of pyknotic cells in CA1 [%; Mean ± SD]	25 ± 17	38 ± 21	0.11
CA1 cell layer - normalized surface/length [mm2/mm; Median (IQR)]	.060 (.054 - .075)	.053 (.052 - .062)	0.10
Fluoro Jade [numbers of degenerating cells per mm; Mean ± SD]	94 ± 47	128 ± 37	0.08

No difference between the (p) and the (c) group was found in the behavior score, the neuro-deficit score and the tape-removal-test. The open field and the water maze test did not reveal a difference between the groups either.

## Conclusions

In this 8-min CA model with only mild neurobehavioral damage pifithrin- µ does not bring any clinical benefit despite a trend towards less histological damage. Further studies with longer cardiac arrest times (more severe neuronal damage), different dosages and application routes of pifithrin- µ are planned.
